# Supplementary material for: A Likelihood Approach for Real-Time Calibration of Stochastic Compartmental Epidemic Models
Source: PLoS Comput Biol. 2017 Jan 17;13(1):e1005257. doi: 10.1371/journal.pcbi.1005257 (PMC5240920; doi:10.1371/journal.pcbi.1005257)
Supplement: S1 File — (TAR.GZ) [file pcbi.1005257.s014.tar.gz › HSPH_Online-SI-Revision/IPoi09/n100-severe/n100-severe_BENCH_table0.pdf]

| | | | | | | | | | | | | | | | | | | | | | | | | | | | |
|---|---|---|---|---|---|---|---|---|---|---|---|---|---|---|---|---|---|---|---|---|---|---|---|---|---|---|---|
| 1 | R0 | {1.65278, 9.06793} | {2.86095, 19.9929} | {{2.86095, 19.9929}, {2.86095, 19.9929}} | {73.0993, 120.479} | inf | 9005. | 24 981.1 | {21 454., 28 222.} | 180.959 | 0.450155 | newly | 6768. | 8594.1 | {6966., 9842.} | 33.3043 | 0.136983 | Reff | 0.958398 | 1.03467 | {0.58927, 1.61827} | 37.6349 | ar | 66 342. | 93 033. | {92 897., 93 188.} | 40.2324 |
| 2 | R0 | {1.4321, 3.80644} | {2.93502, 12.6864} | {{2.93502, 12.6864}, {2.93502, 12.6864}} | {104.945, 233.288} | inf | 4783. | 25 098.2 | {21 562., 29 164.} | 424.737 | 0.704629 | newly | 7700. | 13 011.4 | {10 177., 15 506.} | 70.7737 | 0.22713 | Reff | 0.875729 | 0.911573 | {0.494433, 1.62075} | 39.5559 | ar | 54 217. | 93 553.6 | {93 423., 93 708.} | 72.554 |
| 3 | R0 | {1.68388, 4.76028} | {2.93653, 10.872} | {{2.93653, 10.872}, {2.93653, 10.872}} | {74.3907, 128.39} | inf | 8327. | 22 491.1 | {16 377., 28 242.} | 170.099 | 0.422237 | newly | 10 200. | 12 848.9 | {9096., 17 367.} | 31.1416 | 0.112377 | Reff | 0.78489 | 0.674098 | {0.380369, 1.23513} | 42.9962 | ar | 68 299. | 93 586.3 | {93 426., 93 765.} | 37.0244 |
| 4 | R0 | {1.66014, 6.95383} | {2.95209, 14.037} | {{2.95209, 14.037}, {2.95209, 14.037}} | {77.8217, 101.86} | inf | 9062. | 22 870.9 | {17 309., 27 938.} | 152.383 | 0.395029 | newly | 8732. | 10 471. | {7739., 13 120.} | 24.8916 | 0.0934877 | Reff | 0.943906 | 0.693276 | {0.387196, 1.34795} | 48.7187 | ar | 66 820. | 93 710.9 | {93 555., 93 857.} | 40.2438 |
| 5 | R0 | {1.69984, 11.2038} | {2.53741, 16.1789} | {{2.53741, 16.1789}, {2.53741, 16.1789}} | {49.2735, 44.4055} | inf | 9747. | 18 496.7 | {13 959., 23 298.} | 91.7567 | 0.276945 | newly | 5980. | 7459.74 | {5321., 9695.} | 31.9151 | 0.120724 | Reff | 0.958166 | 0.713409 | {0.45341, 1.08185} | 40.0873 | ar | 68 462. | 89 740. | {89 517., 89 990.} | 31.0801 |
| 6 | R0 | {1.65876, 6.66217} | {2.86001, 13.5542} | {{2.86001, 13.5542}, {2.86001, 13.5542}} | {72.4186, 103.45} | inf | 9053. | 22 403.5 | {16 365., 27 756.} | 148.354 | 0.386247 | newly | 9118. | 10 696.5 | {7205., 13 973.} | 26.3667 | 0.102598 | Reff | 1.0042 | 0.760428 | {0.401889, 1.19034} | 45.7656 | ar | 67 105. | 92 998.7 | {92 780., 93 186.} | 38.5868 |
| 7 | R0 | {1.69966, 5.89996} | {2.79531, 12.1249} | {{2.79531, 12.1249}, {2.79531, 12.1249}} | {64.4629, 105.508} | inf | 10 219. | 24 183.1 | {19 099., 27 445.} | 140.136 | 0.376846 | newly | 11 408. | 13 365.9 | {10 056., 15 535.} | 24.4926 | 0.0995176 | Reff | 0.93415 | 0.99468 | {0.549111, 1.52015} | 35.1287 | ar | 69 396. | 92 457.4 | {92 317., 92 603.} | 33.2315 |
| 8 | R0 | {1.53428, 6.3734} | {2.8833, 17.1847} | {{2.8833, 17.1847}, {2.8833, 17.1847}} | {87.9253, 169.632} | inf | 6765. | 24 646.6 | {16 842., 28 451.} | 267.733 | 0.554483 | newly | 7091. | 9895.8 | {8117., 11 584.} | 46.9815 | 0.176759 | Reff | 0.973639 | 1.10214 | {0.561465, 2.0688} | 40.9845 | ar | 59 855. | 93 197.4 | {93 009., 93 386.} | 55.7052 |
| 9 | R0 | {1.58939, 3.48608} | {2.9884, 12.8326} | {{2.9884, 12.8326}, {2.9884, 12.8326}} | {88.0218, 268.11} | inf | 7643. | 26 165.5 | {17 664., 29 912.} | 246.321 | 0.533918 | newly | 13 814. | 14 362.5 | {11 895., 16 179.} | 15.4337 | 0.0797438 | Reff | 0.959229 | 1.2553 | {0.717485, 2.11393} | 44.4947 | ar | 62 575. | 93 983.5 | {93 851., 94 123.} | 50.1934 |
| 10 | R0 | {1.58528, 5.5593} | {2.93978, 19.9817} | {{2.93978, 19.9817}, {2.93978, 19.9817}} | {85.4423, 259.428} | inf | 7290. | 25 201.7 | {16 725., 29 226.} | 251.091 | 0.538573 | newly | 8668. | 8775.13 | {7077., 10 205.} | 16.8967 | 0.0957196 | Reff | 0.933207 | 1.1584 | {0.625526, 2.10659} | 46.2163 | ar | 63 039. | 93 627.3 | {93 451., 93 834.} | 48.5228 |
| 11 | R0 | {1.58305, 4.62721} | {2.9884, 12.8326} | {{2.9884, 12.8326}, {2.9884, 12.8326}} | {88.7748, 177.329} | inf | 7294. | 25 608.5 | {20 203., 29 739.} | 252.373 | 0.540524 | newly | 9915. | 12 970.3 | {9877., 15 724.} | 34.2554 | 0.127069 | Reff | 0.910428 | 0.842733 | {0.473303, 1.45882} | 39.4468 | ar | 63 222. | 93 983.5 | {93 851., 94 123.} | 48.6563 |
| 12 | R0 | {1.70234, 9.34626} | {2.7811, 19.6712} | {{2.7811, 19.6712}, {2.7811, 19.6712}} | {63.3692, 110.471} | inf | 9412. | 23 837.8 | {17 336., 27 262.} | 160.944 | 0.428096 | newly | 6925. | 8434.57 | {6909., 9696.} | 31.639 | 0.14314 | Reff | 0.976973 | 1.13235 | {0.663598, 1.80046} | 38.2338 | ar | 68 213. | 92 343.4 | {92 163., 92 489.} | 35.3751 |
| 13 | R0 | {1.68556, 9.74954} | {2.59602, 16.9461} | {{2.59602, 16.9461}, {2.59602, 16.9461}} | {54.0153, 73.8144} | inf | 9667. | 18 747.1 | {13 263., 24 184.} | 98.6864 | 0.295171 | newly | 6771. | 7320.48 | {4951., 10 010.} | 25.9817 | 0.113552 | Reff | 0.938014 | 0.802065 | {0.427097, 1.35079} | 44.7489 | ar | 68 426. | 90 461.1 | {90 244., 90 674.} | 32.2028 |
| 14 | R0 | {1.59876, 9.78897} | {2.52229, 17.4218} | {{2.53519, 17.5902}, {2.53519, 17.5902}} | {57.7652, 77.9733} | inf | 7640. | 19 672.9 | {11 314., 23 609.} | 164.734 | 0.42588 | newly | 5391. | 7682.69 | {5226., 9421.} | 51.9302 | 0.195978 | Reff | 1.0474 | 1.01552 | {0.561494, 1.83946} | 38.2774 | ar | 63 553. | 89 602.5 | {89 378., 89 957.} | 40.9886 |
| 15 | R0 | {1.43577, 5.12819} | {2.93978, 19.9817} | {{2.93978, 19.9817}, {2.93978, 19.9817}} | {104.753, 289.644} | inf | 5339. | 25 201.7 | {16 725., 29 226.} | 375.793 | 0.660536 | newly | 6966. | 8775.13 | {7077., 10 205.} | 37.0778 | 0.158547 | Reff | 0.97842 | 1.1584 | {0.625526, 2.10659} | 43.5302 | ar | 53 259. | 93 627.3 | {93 451., 93 834.} | 75.7962 |
| 16 | R0 | {1.58866, 8.73191} | {2.86095, 19.9929} | {{2.86095, 19.9929}, {2.86095, 19.9929}} | {80.0857, 128.964} | inf | 7934. | 24 981.1 | {21 454., 28 222.} | 218.001 | 0.50154 | newly | 6295. | 8594.1 | {6966., 9842.} | 42.3352 | 0.164322 | Reff | 1.05624 | 1.03467 | {0.58927, 1.61827} | 35.7914 | ar | 64 349. | 93 033. | {92 897., 93 188.} | 44.5756 |
| 17 | R0 | {1.43012, 3.8163} | {2.8253, 12.8018} | {{2.82285, 12.7912}, {2.82285, 12.7912}} | {97.5567, 235.452} | inf | 5239. | 23 562.1 | {14 152., 27 741.} | 355.327 | 0.648363 | newly | 8949. | 12 899.6 | {9272., 14 995.} | 53.2065 | 0.201914 | Reff | 0.975428 | 1.22314 | {0.629496, 2.0154} | 44.4102 | ar | 53 353. | 92 702.4 | {92 491., 92 888.} | 73.753 |
| 18 | R0 | {1.47965, 5.16472} | {2.90253, 14.8815} | {{2.90379, 14.8888}, {2.90379, 14.8888}} | {96.1636, 188.138} | inf | 5515. | 24 872.6 | {20 442., 28 721.} | 352.317 | 0.637603 | newly | 7086. | 11 298. | {8642., 13 293.} | 64.1852 | 0.216626 | Reff | 0.947154 | 1.01361 | {0.514784, 1.6567} | 40.2103 | ar | 56 086. | 93 344.7 | {93 182., 93 534.} | 66.4314 |
| 19 | R0 | {1.57191, 5.2314} | {2.86001, 13.5542} | {{2.86001, 13.5542}, {2.86001, 13.5542}} | {81.9449, 159.093} | inf | 6751. | 22 403.5 | {16 365., 27 756.} | 231.922 | 0.509494 | newly | 7954. | 10 696.5 | {7205., 13 973.} | 38.424 | 0.136011 | Reff | 0.829937 | 0.760428 | {0.401889, 1.19034} | 43.2696 | ar | 63 119. | 92 998.7 | {92 780., 93 186.} | 47.3386 |
| 20 | R0 | {1.54435, 5.32911} | {2.8833, 17.1847} | {{2.8833, 17.1847}, {2.8833, 17.1847}} | {86.6999, 222.468} | inf | 6968. | 24 646.6 | {16 842., 28 451.} | 257.306 | 0.542906 | newly | 8356. | 9895.8 | {8117., 11 584.} | 27.5867 | 0.11882 | Reff | 0.898704 | 1.10214 | {0.561465, 2.0688} | 44.6061 | ar | 61 870. | 93 197.4 | {93 009., 93 386.} | 50.6342 |
| 21 | R0 | {1.57932, 4.90031} | {2.79531, 12.1249} | {{2.79531, 12.1249}, {2.79531, 12.1249}} | {76.9945, 147.431} | inf | 8141. | 21 575. | {14 783., 26 387.} | 165.017 | 0.4153 | newly | 10 527. | 11 241.7 | {8018., 14 230.} | 18.4516 | 0.0773902 | Reff | 0.930156 | 0.693966 | {0.403671, 0.992223} | 43.2117 | ar | 63 595. | 92 457.4 | {92 317., 92 603.} | 45.3846 |
| 22 | R0 | {1.47044, 4.07694} | {2.76203, 15.0558} | {{2.76203, 15.0558}, {2.76203, 15.0558}} | {87.837, 269.292} | inf | 5758. | 22 800.3 | {13 799., 26 705.} | 296.677 | 0.578349 | newly | 9422. | 10 420.8 | {6913., 12 413.} | 22.1759 | 0.0976974 | Reff | 0.991724 | 1.08368 | {0.55097, 1.97339} | 42.1806 | ar | 55 895. | 92 152. | {91 982., 92 321.} | 64.8663 |
| 23 | R0 | {1.64997, 3.34546} | {2.82466, 9.2843} | {{2.82466, 9.2843}, {2.82466, 9.2843}} | {71.1946, 177.519} | inf | 6829. | 20 687.4 | {14 746., 25 863.} | 203.056 | 0.46964 | newly | 10 882. | 13 434.6 | {8686., 18 073.} | 31.2821 | 0.115316 | Reff | 0.75727 | 0.646849 | {0.379521, 0.912958} | 41.5559 | ar | 66 404. | 92 686.6 | {92 521., 92 865.} | 39.5798 |
| 24 | R0 | {1.67405, 4.50254} | {2.98388, 9.60029} | {{2.98388, 9.60029}, {2.98388, 9.60029}} | {78.2432, 113.219} | inf | 9359. | 25 507. | {20 276., 29 672.} | 173.563 | 0.428839 | newly | 12 907. | 16 700.4 | {12 737., 20 610.} | 32.2997 | 0.118076 | Reff | 0.908708 | 0.840481 | {0.469901, 1.46989} | 39.2752 | ar | 68 017. | 93 948. | {93 795., 94 112.} | 38.1243 |
| 25 | R0 | {1.50663, 3.1286} | {2.98388, 9.60029} | {{2.98388, 9.60029}, {2.98388, 9.60029}} | {98.05, 206.856} | inf | 5849. | 25 507. | {20 276., 29 672.} | 336.091 | 0.627641 | newly | 11 133. | 16 700.4 | {12 737., 20 610.} | 51.0938 | 0.173174 | Reff | 0.89994 | 0.840481 | {0.469901, 1.46989} | 39.1483 | ar | 58 823. | 93 948. | {93 795., 94 112.} | 59.713 |
| 26 | R0 | {1.69822, 6.34992} | {2.76203, 15.0558} | {{2.76203, 15.0558}, {2.76203, 15.0558}} | {62.6426, 137.102} | inf | 9988. | 22 800.3 | {13 799., 26 705.} | 133.173 | 0.363784 | newly | 10 139. | 10 420.8 | {6913., 12 413.} | 17.3549 | 0.0830063 | Reff | 0.906629 | 1.08368 | {0.55097, 1.97339} | 45.3438 | ar | 69 816. | 92 152. | {91 982., 92 321.} | 31.9927 |
| 27 | R0 | {1.55869, 4.92988} | {2.99911, 15.6207} | {{2.99911, 15.6207}, {2.99911, 15.6207}} | {92.4122, 216.858} | inf | 7340. | 25 679.1 | {19 173., 29 943.} | 252.001 | 0.53278 | newly | 9500. | 11 188.2 | {8490., 13 302.} | 27.1667 | 0.114066 | Reff | 0.908124 | 1.04934 | {0.511978, 2.02794} | 45.2163 | ar | 62 344. | 94 013.6 | {93 846., 94 150.} | 50.7982 |
| 28 | R0 | {1.46085, 6.06899} | {2.32891, 10.9956} | {{2.32891, 10.9956}, {2.32891, 10.9956}} | {59.4216, 81.1768} | inf | 5497. | 16 385.6 | {11 780., 20 467.} | 200.611 | 0.474509 | newly | 5894. | 9710.05 | {6666., 12 818.} | 67.7765 | 0.219047 | Reff | 0.925463 | 0.818008 | {0.497455, 1.39651} | 35.6549 | ar | 55 484. | 86 722.2 | {86 521., 86 964.} | 56.3013 |
| 29 | R0 | {1.63017, 6.25164} | {2.81617, 13.9425} | {{2.81617, 13.9425}, {2.81617, 13.9425}} | {72.7531, 123.021} | inf | 8283. | 23 533.7 | {16 964., 27 701.} | 184.79 | 0.443831 | newly | 8506. | 11 256.6 | {8411., 13 693.} | 36.2238 | 0.131658 | Reff | 0.896577 | 0.91297 | {0.500687, 1.43884} | 39.3743 | ar | 66 231. | 92 631.6 | {92 473., 92 787.} | 39.8614 |
| 30 | R0 | {1.45173, 5.68165} | {2.59602, 16.9461} | {{2.59602, 16.9461}, {2.59602, 16.9461}} | {78.8225, 198.26} | inf | 5301. | 20 428.7 | {12 946., 24 544.} | 294.036 | 0.596831 | newly | 6273. | 8419.98 | {6513., 10 116.} | 46.8321 | 0.202158 | Reff | 0.953496 | 1.18684 | {0.631041, 1.99987} | 45.0519 | ar | 55 280. | 90 461.1 | {90 244., 90 674.} | 63.6416 |
| 31 | R0 | {1.55808, 2.77316} | {2.9409, 8.83507} | {{2.77394, 8.24535}, {2.9751, 8.95585}} | {88.7517, 218.592} | inf | 4924. | 22 197.8 | {15 201., 28 696.} | 350.808 | 0.638863 | newly | 9080. | 15 134.4 | {9438., 21 111.} | 67.9769 | 0.210191 | Reff | 0.782265 | 0.676288 | {0.342672, 1.18698} | 43.0431 | ar | 60 940. | 93 555.7 | {92 184., 94 006.} | 53.521 |
| 32 | R0 | {1.56797, 3.90327} | {2.70795, 10.355} | {{2.70795, 10.355}, {2.70795, 10.355}} | {72.7042, 165.29} | inf | 6996. | 22 645. | {18 154., 26 217.} | 223.685 | 0.501341 | newly | 11 191. | 14 110.2 | {10 528., 17 129.} | 29.705 | 0.110603 | Reff | 0.933679 | 0.858422 | {0.494445, 1.22849} | 34.2662 | ar | 62 228. | 91 627.4 | {91 451., 91 807.} | 47.2447 |
| 33 | R0 | {1.70027, 5.43261} | {2.93653, 10.872} | {{2.93653, 10.872}, {2.93653, 10.872}} | {72.7096, 100.125} | inf | 8804. | 22 491.1 | {16 377., 28 242.} | 155.465 | 0.398045 | newly | 10 094. | 12 848.9 | {9096., 17 367.} | 32.1744 | 0.115426 | Reff | 0.817558 | 0.674098 | {0.380369, 1.23513} | 43.6901 | ar | 68 798. | 93 586.3 | {93 426., 93 765.} | 36.0306 |
| 34 | R0 | {1.61668, 6.86192} | {2.7811, 19.6712} | {{2.7811, 19.6712}, {2.7811, 19.6712}} | {72.0254, 186.672} | inf | 8157. | 23 837.8 | {17 336., 27 262.} | 199.04 | 0.48196 | newly | 7767. | 8434.57 | {6909., 9696.} | 20.6682 | 0.108415 | Reff | 0.928411 | 1.13235 | {0.663598, 1.80046} | 40.5314 | ar | 64 693. | 92 343.4 | {92 163., 92 489.} | 42.7409 |
| 35 | R0 | {1.55955, 3.45071} | {2.93653, 10.872} | {{2.93653, 10.872}, {2.93653, 10.872}} | {88.2934, 215.066} | inf | 6759. | 25 316.4 | {19 236., 29 202.} | 275.445 | 0.560344 | newly | 11 360. | 15 638.3 | {11 973., 18 530.} | 41.6499 | 0.151758 | Reff | 0.876124 | 1.05036 | {0.56907, 1.97379} | 46.2798 | ar | 61 783. | 93 586.3 | {93 426., 93 765.} | 51.4758 |
| 36 | R0 | {1.65068, 4.91307} | {2.99939, 13.7076} | {{2.99939, 13.7076}, {2.99939, 13.7076}} | {81.7063, 179.003} | inf | 9077. | 26 614.3 | {19 727., 30 091.} | 197.038 | 0.470982 | newly | 12 338. | 13 392.9 | {11 468., 15 145.} | 17.323 | 0.0820583 | Reff | 0.997374 | 1.14294 | {0.618084, 2.01772} | 37.5905 | ar | 66 453. | 94 036.2 | {93 909., 94 200.} | 41.5079 |
| 37 | R0 | {1.46131, 3.06714} | {2.93906, 10.3908} | {{2.93906, 10.3908}, {2.93906, 10.3908}} | {101.125, 238.778} | inf | 5039. | 24 894.3 | {19 267., 29 024.} | 394.033 | 0.683369 | newly | 9251. | 15 223. | {11 702., 18 531.} | 65.0269 | 0.210455 | Reff | 0.841612 | 0.829414 | {0.473923, 1.31408} | 37.4699 | ar | 56 452. | 93 608.6 | {93 494., 93 734.} | 65.8197 |
| 38 | R0 | {1.71034, 5.2546} | {2.9884, 12.8326} | {{2.9884, 12.8326}, {2.9884, 12.8326}} | {74.7255, 144.216} | inf | 9467. | 25 608.5 | {20 203., 29 739.} | 172.232 | 0.43093 | newly | 11 210. | 12 970.3 | {9877., 15 724.} | 22.3691 | 0.0903673 | Reff | 0.857787 | 0.842733 | {0.473303, 1.45882} | 39.2127 | ar | 69 816. | 93 983.5 | {93 851., 94 123.} | 34.616 |
| 39 | R0 | {1.43565, 5.57989} | {2.44101, 12.0941} | {{2.44101, 12.0941}, {2.44101, 12.0941}} | {70.0282, 116.744} | inf | 5165. | 16 651. | {10 788., 22 028.} | 224.28 | 0.497945 | newly | 6041. | 8832.73 | {5302., 12 518.} | 52.8316 | 0.179023 | Reff | 0.90825 | 0.725189 | {0.420025, 1.17901} | 38.9406 | ar | 54 407. | 88 460.4 | {88 209., 88 708.} | 62.5902 |
| 40 | R0 | {1.40327, 4.00764} | {2.2983, 8.81055} | {{2.2983, 8.81055}, {2.2983, 8.81055}} | {63.7817, 119.844} | inf | 4452. | 16 540.8 | {11 703., 20 182.} | 274.483 | 0.571219 | newly | 7170. | 12 391. | {8368., 15 657.} | 77.0339 | 0.248286 | Reff | 0.942464 | 0.934738 | {0.541296, 1.5485} | 34.2329 | ar | 51 976. | 86 210.8 | {85 948., 86 504.} | 65.8665 |
| 41 | R0 | {1.63612, 4.26741} | {2.82493, 9.28789} | {{2.82466, 9.2843}, {2.82466, 9.2843}} | {72.6602, 117.647} | inf | 7141. | 20 444.7 | {14 167., 25 845.} | 186.567 | 0.445159 | newly | 9905. | 13 258.4 | {8674., 18 306.} | 39.1559 | 0.136876 | Reff | 0.840131 | 0.640557 | {0.366839, 1.0747} | 45.5988 | ar | 65 029. | 92 693.8 | {92 546., 92 885.} | 42.5422 |
| 42 | R0 | {1.57426, 6.50271} | {2.97157, 19.3547} | {{2.97157, 19.3547}, {2.97157, 19.3547}} | {88.7598, 197.641} | inf | 7623. | 25 013.6 | {14 529., 29 699.} | 234.018 | 0.517919 | newly | 7814. | 9066.24 | {6436., 10 612.} | 28.9375 | 0.136315 | Reff | 0.952711 | 1.24103 | {0.607954, 2.27456} | 49.8816 | ar | 62 522. | 93 849.1 | {93 703., 93 993.} | 50.1057 |
| 43 | R0 | {1.37818, 5.37636} | {2.59602, 16.9461} | {{2.59602, 16.9461}, {2.59602, 16.9461}} | {88.3658, 215.197} | inf | 4296. | 20 428.7 | {12 946., 24 544.} | 382.803 | 0.674876 | newly | 5382. | 8419.98 | {6513., 10 116.} | 68.318 | 0.257384 | Reff | 0.94794 | 1.18684 | {0.631041, 1.99987} | 45.3997 | ar | 50 098. | 90 461.1 | {90 244., 90 674.} | 80.5682 |
| 44 | R0 | {1.66606, 4.92641} | {2.96796, 12.4643} | {{2.96796, 12.4643}, {2.96796, 12.4643}} | {78.1424, 153.01} | inf | 9374. | 25 584.5 | {17 657., 29 593.} | 178.95 | 0.444176 | newly | 12 427. | 14 094.2 | {11 478., 16 386.} | 23.7681 | 0.108252 | Reff | 0.958134 | 1.16618 | {0.645353, 2.08855} | 43.976 | ar | 67 066. | 93 827.4 | {93 696., 93 953.} | 39.903 |
| 45 | R0 | {1.623, 5.9449} | {2.86001, 13.5542} | {{2.86001, 13.5542}, {2.86001, 13.5542}} | {76.2175, 127.997} | inf | 8468. | 24 182. | {20 636., 28 065.} | 188.815 | 0.454412 | newly | 9497. | 12 127.1 | {9217., 14 418.} | 33.9838 | 0.132523 | Reff | 0.985843 | 1.0564 | {0.545261, 1.73337} | 41.7067 | ar | 64 554. | 92 998.7 | {92 780., 93 186.} | 44.0634 |
| 46 | R0 | {1.65235, 5.51234} | {2.96947, 15.963} | {{2.96947, 15.963}, {2.96947, 15.963}} | {79.7119, 189.587} | inf | 8716. | 25 738. | {14 963., 29 503.} | 201.226 | 0.480487 | newly | 10 647. | 11 185.9 | {8356., 12 874.} | 19.0759 | 0.102459 | Reff | 0.988634 | 1.17321 | {0.599684, 2.23384} | 41.1912 | ar | 66 509. | 93 829. | {93 704., 93 974.} | 41.0771 |
| 47 | R0 | {1.62138, 8.82208} | {2.67299, 16.6779} | {{2.67299, 16.6779}, {2.67299, 16.6779}} | {64.8589, 89.0473} | inf | 8399. | 21 855.9 | {15 146., 25 734.} | 163.251 | 0.412159 | newly | 6515. | 8803.63 | {5601., 10 701.} | 42.6779 | 0.158307 | Reff | 0.936704 | 0.91657 | {0.514604, 1.38573} | 35.9358 | ar | 65 598. | 91 280.8 | {91 103., 91 497.} | 39.1519 |
| 48 | R0 | {1.64073, 6.69261} | {2.68687, 12.2271} | {{2.68687, 12.2271}, {2.68687, 12.2271}} | {63.7606, 82.6955} | inf | 8727. | 22 498.5 | {17 188., 26 008.} | 159.77 | 0.407115 | newly | 8793. | 12 218.6 | {8849., 14 776.} | 42.4977 | 0.151494 | Reff | 0.968375 | 0.932351 | {0.545354, 1.54009} | 33.6737 | ar | 66 438. | 91 401.6 | {91 175., 91 601.} | 37.5743 |
| 49 | R0 | {1.65556, 3.796} | {2.98388, 9.60029} | {{2.98388, 9.60029}, {2.98388, 9.60029}} | {80.2339, 152.905} | inf | 8332. | 25 507. | {20 276., 29 672.} | 206.884 | 0.477691 | newly | 12 770. | 16 700.4 | {12 737., 20 610.} | 33.4768 | 0.121664 | Reff | 0.845809 | 0.840481 | {0.469901, 1.46989} | 38.643 | ar | 66 575. | 93 948. | {93 795., 94 112.} | 41.116 |
| 50 | R0 | {1.56828, 3.65713} | {2.82466, 9.2843} | {{2.82466, 9.2843}, {2.82466, 9.2843}} | {80.112, 153.868} | inf | 6216. | 20 687.4 | {14 746., 25 863.} | 232.808 | 0.509944 | newly | 9733. | 13 434.6 | {8686., 18 073.} | 41.879 | 0.143734 | Reff | 0.821841 | 0.646849 | {0.379521, 0.912958} | 43.807 | ar | 62 761. | 92 686.6 | {92 521., 92 865.} | 47.6818 |
